# Pathogen-oriented targeted next-generation sequencing as an adjunctive tool for detection of pathogens in endophthalmitis: a retrospective clinical evaluation

**DOI:** 10.3389/fmed.2026.1776006

**Published:** 2026-04-15

**Authors:** Xianliang Zhang, Xiaoxi Yang, Huayu Wu, Xiaoli Li, Junjun Han, Tianyi Cai, Sheng Zeng, Hongling Chen

**Affiliations:** 1 Henan Eye Hospital, Henan Provincial People’s Hospital, People's Hospital of Zhengzhou University, Zhengzhou, China; 2 School of Nursing and Health, Nanfang College, Guangzhou, China

**Keywords:** culture, endophthalmitis, pathogen identification, positivity rate, tNGS

## Abstract

**Background:**

Endophthalmitis represents one of the most severe ophthalmic emergencies, requiring prompt etiological identification to enable tailored antibiotic administration. Conventional identification methods, such as ocular sample culture, exhibit limited positivity in endophthalmitis, necessitating more advanced approaches.

**Methods:**

The performance of pathogen-oriented targeted next-generation sequencing (tNGS), designed to capture a predefined panel of 207 clinically relevant pathogens, was retrospectively evaluated in 43 patients with endophthalmitis.

**Results:**

The positivity rate of culture was 22.2% (8/36, 95% CI: 10.1–39.2%), whereas that of tNGS was 86.0% (37/43, 95% CI, 72.1–94.7%). tNGS had a turnaround time of less than 24 h, facilitated the detection of polymicrobial infection, and identified microbes in 91.7% (11/12) of specimens that were negative for both smear and culture. Among the 36 paired specimens subjected to both culture and tNGS, tNGS achieved microbial identification in 25 of 28 culture-negative specimens (89.3%), and its positivity rate (88.9%, 32/36, 95% CI, 73.9–96.9%) was significantly higher than that of culture (*p* < 0.001), with the difference being 66.7% (95% CI, 44.4–80.6%). Poor agreement between tNGS and culture was observed (Kappa = −0.009, *p* = 0.887). The positivity rate of tNGS was 100% (9/9) for aqueous humour specimens, and 85.2% (23/27) for vitreous specimens. Additionally, there was no statistically significant difference in the positivity rate of tNGS among endogenous, post-traumatic, and post-operative endophthalmitis (*p* = 0.666). The application of tNGS impacted therapeutic strategy selection and contributed to the control of infections in cases caused by uncommon pathogens (e.g., mycobacterial infection), in endogenous endophthalmitis, and particularly in fungal endophthalmitis.

**Conclusion:**

The higher positivity and shorter turnaround time of pathogen-oriented tNGS render it an adjunctive tool for enhancing pathogen identification in endophthalmitis. We recommend the routine application of tNGS as a valuable complement to culture, in order to facilitate and expedite pathogen detection, thereby advancing efforts toward tailored precision medicine in the management of endophthalmitis.

## 1 Introduction

Endophthalmitis, an infectious inflammation involving the inner coats of the eye, represents one of the most severe ophthalmic emergencies that requires prompt medical intervention. Expedited and accurate etiological identification is pivotal to improving prognosis, as it prevents mistreatment and severe visual impairment (1). However, etiological identification can be challenging due to multiple factors, such as that a broad spectrum of pathogens, including both bacteria and fungi, can cause the disease, and that the microbial profile is likely complicated by the disease type and geographic location (1–3).

Notably, the dominant pathogens vary with the type of endophthalmitis. For postoperative and posttraumatic endophthalmitis, gram-positive cocci, particularly *Staphylococcus epidermidis*, are dominant microbes (3–6). In contrast, gram-negative bacteria (e.g., *Pseudomonas* spp.), represent the major pathogens detected in endogenous endophthalmitis (4). For keratitis-associated endophthalmitis, *Fusarium* infections are frequently observed (3). Additionally, the proportion of fungal infections in endogenous endophthalmitis has been reported to be higher in Western countries than in Asian countries (4), highlighting the impact of geographic and/or ethnic factors on the microbial spectrum.

Conventional methods for pathogen detection in endophthalmitis include microbiological culture of ocular samples, i.e., aqueous humour (AH) or vitreous humour (VH). The method exhibits limited positivity rates ranging from 30 to 50% (3, 7–9), which hinders clinical decision-making. The positivity rate can be compromised by factors such as prior antimicrobial usage (10). More advanced diagnostic approaches, e.g., next-generation sequencing (NGS), have emerged and are being increasingly applied in clinical practice. For instance, the application of an unbiased NGS has facilitated actionable diagnosis of neuroleptospirosis and ensured efficacious antimicrobial therapy (11).

Two NGS strategies, i.e., metagenomic NGS (mNGS) and targeted NGS (tNGS), have been explored for the diagnosis of infectious diseases (12). Specifically, the diagnostic performance of mNGS has been demonstrated in intraocular infections and infectious keratitis, with positivity rates superior to those of culture (13, 14). Compared with unbiased mNGS, the tNGS approach, characterized by target enrichment via PCR-based amplicon sequencing or probe capture-based methods, exhibits advantages in data interpretation, efficiency, cost-effectiveness and detection sensitivity (12, 15). In Henan Province, China, the cost per sample is approximately 3,500 RMB for mNGS, whereas that for tNGS is reduced to 800 RMB.

Previous studies have investigated the performance of a tNGS approach targeting the bacterial 16S rRNA gene and fungal internal transcribed spacer (ITS) region in suspected endophthalmitis. The approach exhibited significantly higher sensitivity and specificity compared with conventional microbial testing (9, 16). Additionally, the application of nanopore targeted sequencing (NTS) has facilitated the detection of polymicrobial infections in suspected endophthalmitis (16). However, to the best of our knowledge, the diagnostic value and performance of tNGS targeting a predefined panel of pathogens remain to be investigated in endophthalmitis, despite promising results reported in other clinical settings (e.g., pulmonary, central nervous system, and bloodstream infections) (17–19).

Herein, we retrospectively evaluated the performance of tNGS, based on amplicon sequencing and targeting 207 clinically relevant pathogens, in etiological identification among 43 cases of suspected endophthalmitis, to further assess the feasibility and reliability of this approach. Our results showed that relative to culture, tNGS exhibited a significantly higher positivity rate, facilitated pathogen detection in culture-negative specimens, enabled polymicrobial identification, and substantially shortened the detection turnaround time. The performance of tNGS may be not compromised when using AH specimens, and does not seem to differ significantly among endogenous, post-traumatic and post-operative endophthalmitis. The study supports the clinical application of pathogen-oriented tNGS, as a complementary tool, for enhanced pathogen identification in suspected endophthalmitis.

## 2 Materials and methods

### 2.1 Patients, clinical specimens and ethics

From August 2023 to August 2024, a total of 43 consecutive patients with suspected endophthalmitis, admitted to the Ocular Trauma Center of Henan Eye Hospital, were enrolled in the study, including 25 males and 18 females. The medical records of all patients were reviewed. Clinical specimens, including 31 VH and 12 AH samples, were analysed using smear, microbiological culture, and/or the tNGS method. Particularly, except for 5 specimens with insufficient sample volumes (e.g., less than 0.1 mL), all other specimens collected during the period were subjected to tNGS. This study was conducted in accordance with the 1964 Declaration of Helsinki. The study protocol was approved by the Ethics Committee of the First Affiliated Hospital of Zhengzhou University [approval number HNEEC-2025 (37)]. Written informed consent was obtained from all patients, explicitly informing that the clinical data would be used solely for scientific research purposes.

### 2.2 Clinical sample collection

To avoid contamination, intraocular fluid specimens (VH or AH) were collected under sterile conditions. The AH samples were obtained through slit-lamp-guided anterior chamber paracentesis using 1-mL disposable sterilized syringes in a UV-sterilized environment, whereas VH samples were obtained either via vitreous tap or pars plana vitrectomy under a surgical microscope. All operations were performed by the same operator, and purulent lesions and inflammatory exudates were removed as much as possible during specimen collection. The collected samples were placed in sterile EP tubes and stored at 2–8 °C prior to laboratory analysis.

### 2.3 Conventional microbial testing

Gram staining and KOH mounts were performed on AH or VH specimens. Additionally, the specimens were inoculated onto Columbia blood agar basal medium for bacterial growth and Sabouraud glucose agar for fungal cultivation. For culture-positive cases, the isolated microorganisms were identified using the Vitek 2 Compact automated microbial identification system (bioMérieux, Beijing, China).

### 2.4 DNA extraction

The collected AH and VH specimens were sent, on ice packs, to Practice Medicine Co., Ltd. (https://shijianmed.cn/gywm) for pathogen-targeted tNGS testing. The experimental procedures included genomic DNA extraction, ultra-multiplex PCR-based targeted amplification of pathogen-specific genes, sequencing library construction, and high-throughput sequencing. For DNA extraction, the intraocular fluid samples were centrifuged at 13000 rpm for 10 min, followed by the removal of supernatant. Subsequently, 0.5 μm glass beads (2 g) and cell wall-lysing enzymes were added, to disrupt cell walls and enhance the release of microbial nucleic acids. The sample was homogenized using a TGrinder H24 tissue homogenizer at 6 m/s for 45 s with 30-s intervals, for a total of 7 cycles. After homogenization, the mixture was centrifuged at 12,000 rpm for 1 min at room temperature, and 500 μL of the supernatant was transferred to a 1.5-mL centrifuge tube. Nucleic acid extraction was performed using the TIANGEN nucleic acid extraction kit (Beijing, China), following the manufacturer’s manual. The concentration of the extracted DNA was analyzed by Nanodrop spectrophotometry, and the quality was assessed based on the A260/A230 ratio, showing 2.0–2.2 for all specimens.

### 2.5 Construction of sequencing library

The tNGS approach was capable of detecting a total of 207 clinically prevalent pathogens, including 93 bacteria, 60 viruses, 22 fungi, 16 parasites, and 16 other special pathogens (e.g., mycoplasma and chlamydia), as well as 21 antibiotic resistance genes (Table 1). Non-conserved species-specific sequences were identified using BLAST software based on representative NCBI reference genomes of the target pathogens. Primer sets for pathogen identification were designed targeting these specific sequences, to avoid cross-amplification between closely related species, and synthesized by Sangon Biotech (Shanghai, China). The number of primer pairs ranged from 1 to 18 for each target pathogen, depending on factors such as the pathogenicity, frequency of clinical infection, and inherent characteristics of the pathogen. For validation, the designed primers were pooled and evaluated using clinical isolates, assessing specificity, amplification bias and efficiency, and primer-dimer formation.

**Table 1:** The predefined panel of 207 pathogens and 21 resistance genes for the pathogen-oriented tNGS.

Type	Pathogen list	
Bacteria	G^+^	*Streptococcus pneumoniae*, *Streptococcus pyogenes*, *Streptococcus agalactiae*, *Streptococcus intermedius*, *Streptococcus mitis*, *Streptococcus dysgalactiae*, *Streptococcus anginosus*, *Streptococcus equinus*, *Streptococcus suis*, *Peptostreptococcus anaerobius*, Viridans streptococci, *Streptococcus mutans*, *Streptococcus salivarius*, *Streptococcus infantarius*, *Staphylococcus aureus*, *Staphylococcus epidermidis*, *Staphylococcus haemolyticus*, *Staphylococcus hominis*, *Staphylococcus lugdunensis*, *Staphylococcus capitis*, *Enterococcus faecium*, *Enterococcus faecalis*, *Enterococcus avium*, *Enterococcus gallinarum*, *Bacillus anthracis*, *Bacillus cereus* group, *Clostridium perfringens*, *Clostridium septicum*, *Clostridium tetani*, *Corynebacterium diphtheriae*, *Corynebacterium striatum*, *Lactobacillus iners*, Nocardia spp., *Nocardia abscessus*, *Nocardia asteroides*, *Nocardia brasiliensis*, Nocardia *gelsenkirchenensis*, *Nocardia farcinica*, *Nocardia otitidiscaviarum*, *Tropheryma whipplei*, *Finegoldia magna*, *Listeria monocytogenes*, *Dialister pneumosintes*, *Mycobacterium tuberculosis* complex, *Mycobacterium avium*, *Mycobacterium intracellulare*, *Mycobacterium abscessus*, *Mycobacterium chelonae*, *Mycobacterium kansasii*, *Mycobacterium xenopi*, *Mycobacterium fortuitum*
	G^−^	*Pseudomonas aeruginosa*, *Pseudomonas alcaligenes*, *Acinetobacter baumannii*, *Klebsiella pneumoniae*, *Klebsiella aerogenes*, *Klebsiella oxytoca*, *Escherichia coli*, *Haemophilus influenzae*, *Haemophilus parainfluenzae*, *Moraxella catarrhalis*, *Enterobacter cloacae* complex, *Stenotrophomonas maltophilia*, Burkholderia spp., *Burkholderia cenocepacia*, *Burkholderia cepacia*, *Burkholderia multivorans*, *Legionella pneumophila*, *Bordetella pertussis*, Brucella spp., *Achromobacter xylosoxidans*, *Bacteroides fragilis*, *Campylobacter coli*, *Chryseobacterium indologenes*, *Citrobacter koseri*, *Fusobacterium mortiferum*, *Fusobacterium necrophorum*, *Fusobacterium nucleatum*, *Aggregatibacter aphrophilus*, *Morganella morganii*, *Elizabethkingia meningoseptica*, *Neisseria meningitidis*, *Neisseria gonorrhoeae*, *Pasteurella multocida*, *Proteus mirabilis*, *Proteus vulgaris*, *Salmonella Paratyphi*, *Salmonella Typhi*, *Serratia marcescens*, *Prevotella intermedia*, *Kingella kingae*, *Vibrio vulnificus*, *Capnocytophaga canimorsus*
Viruses	DNA	Human herpesvirus 1, Human herpesvirus 2, Varicella-zoster virus (Human herpesvirus 3), Epstein–Barr virus (Human herpesvirus 4), Cytomegalovirus (Human herpesvirus 5), Human herpesvirus 6, Human herpesvirus 7, Human herpesvirus 8, Human polyomavirus 1 (BK virus), Human polyomavirus 2 (JC virus), Human polyomavirus 3 (KIPyV), Human polyomavirus 4 (WU virus), Parvovirus B19, Human bocavirus, Adenovirus, Human adenovirus B, Pseudorabies virus
	RNA	Human coronavirus 229E, Human coronavirus NL63, Human coronavirus OC43, Novel coronavirus, Middle East respiratory syndrome coronavirus (MERS-CoV), Influenza A virus (H1N1), Influenza A virus (H3N2), Influenza B virus (Victoria), Influenza B virus (Yamagata), Influenza C virus, Human parainfluenza virus 1, Human parainfluenza virus 2, Human parainfluenza virus 3, Human parainfluenza virus 4, Highly pathogenic avian influenza virus (H5N1), Highly pathogenic avian influenza virus (H7N9), Human rhinovirus A, Human rhinovirus B, Human rhinovirus C, Human metapneumovirus, Respiratory syncytial virus, Human coronavirus HKU1, Human parechovirus 1 (HPeV-1), Human parechovirus 3 (HPeV-3), Enterovirus spp., Coxsackievirus A, Coxsackievirus B, Echovirus, Enterovirus D68, Enterovirus B69, Enterovirus D70, Enterovirus A71, Poliovirus, Norovirus, Rotavirus A, Hantavirus, Severe fever with thrombocytopenia syndrome virus, Human immunodeficiency virus, Mumps virus, Chikungunya virus, Dengue virus, Japanese encephalitis virus, Rabies virus
Fungi		*Candida albicans*, *Candida tropicalis*, Candida glabrata, *Candida parapsilosis*, *Candida krusei*, *Candida lusitaniae*, *Aspergillus fumigatus*, Aspergillus flavus, *Aspergillus niger*, Aspergillus terreus, *Cryptococcus neoformans*, Cryptococcus gattii, *Cryptococcus laurentii*, Pneumocystis jirovecii, Rhizomucor pusillus, Rhizopus microsporus, Geotrichum capitatum, Talaromyces marneffei, Scedosporium apiospermum, Trichosporon asahii, Fusarium spp., Histoplasma capsulatum
Parasites		Plasmodium falciparum, Plasmodium malariae, Plasmodium ovale, Plasmodium vivax, *Angiostrongylus cantonensis*, Strongyloides stercoralis, Toxoplasma gondii, *Entamoeba histolytica*, Leishmania, Babesia microti, *Clonorchis sinensis*, Paragonimus westermani, Echinococcus granulosus, Spirometra mansoni, Taenia asiatica, Taenia solium
Special/other pathogens		*Mycoplasma pneumoniae*, *Chlamydia pneumoniae*, *Chlamydia psittaci*, *Chlamydia trachomatis*, *Ureaplasma urealyticum*, *Ureaplasma parvum*, *Mycoplasma hominis*, *Treponema pallidum*, *Borrelia burgdorferi*, *Leptospira interrogans*, *Coxiella burnetii*, *Rickettsia prowazekii*, *Rickettsia rickettsii*, *Rickettsia felis*, *Orientia tsutsugamushi*, *Bartonella henselae*
Resistance gene list		
SHV, mecA, CMY, CTX, GIM, KPC, MCR, MOX-2, NDM-1, NDM-2, OXA-23, OXA-48, VanA, VanB, VanC, katG, pncA, inhA, embB, rpoB, rpsL		

Limit of detection for representative pathogens was determined, and listed as follows: 3.125 CFU/mL for *Staphylococcus aureus*, 6.25 CFU/mL for *Streptococcus pneumoniae*, 1.56 CFU/mL for *Klebsiella pneumoniae*, 0.78 CFU/mL for *Pseudomonas aeruginosa*, 12.5 CFU/mL for *Candida albicans*, and 25 CFU/mL for *Cryptococcus gattii*.

The optimized primer sets were used for the enrichment and amplification of pathogen-specific sequences in VH and AH samples. Amplicons were purified with magnetic beads to remove primer dimers and impurities, followed by a second round of PCR amplification for the ligation of sequencing adapters and addition of distinct barcodes. The quality of the constructed library was assessed using an Agilent 2,100 Bioanalyzer (Agilent Technologies, Santa Clara, CA, USA). The library fragments were approximately 350 bp in length, with no primer dimers or nonspecific amplification products detected. The concentration of the constructed libraries was no less than 1 ng/μL.

### 2.6 Sequencing

Constructed libraries were pooled in equal mass ratios. DNA nanoballs (DNBs) for sequencing were prepared using the DNBSEQ One-step DNB Preparation Kit (Wuhan MGI Tech Co., Ltd., China), followed by high-throughput sequencing performed on the MGISEQ-200 platform with the single-end 75 bp (SE75) sequencing mode. A sequencing data volume of >0.1 million reads was achieved for all specimens analyzed. Additionally, no contamination was detected in negative controls, and no cross-contamination was observed between individual samples. Internal reference controls were detected for all specimens.

### 2.7 Data analysis

The amplicons generated after sequencing were subjected to quality control, and low-quality reads and those shorter than 60 bp were filtered out. The remaining reads were aligned to the human genome to exclude human-derived sequences, yielding high-quality pathogen-derived amplicons. These high-quality amplicons were further mapped back to the predefined targeted regions to identify specific pathogens.

### 2.8 Statistical analysis

Statistical analyses were performed using IBM SPSS Statistics Software Version 26 (SPSS, Inc., Chicago, IL, USA). The confidence interval (CI) around the positivity rates was calculated using exact Clopper–Pearson method. McNemar’s and Kappa test were employed to compare the positivity rates and the binary positivity agreement between tNGS and culture, respectively. The CI around paired positivity rate difference was calculated using the Newcombe method (http://www.vassarstats.net/prop2_ind.html). Fisher’s Exact Test was used to compare positivity rates among endophthalmitis subgroups. A *p*-value of < 0.05 was considered statistically significant.

## 3 Results

### 3.1 Demographic details of patients

Clinical data of 43 patients with suspected endophthalmitis, including 25 males and 18 females, were retrospectively analyzed. Patient ages were normally distributed, with a mean of 54.8 ± 17.9 years (range, 6–90 years). Specifically, the study enrolled 14 cases of endogenous endophthalmitis, 15 cases of post-traumatic endophthalmitis, and 14 cases of post-operative endophthalmitis. A total of 31 VH and 12 AH specimens were detected by smear, culture and/or the tNGS method, depending on individual case conditions (Table 2).

**Table 2:** Demographic details of 43 endophthalmitis patients.

Patient No.	Gender^a^	Age	Laterality^b^	Specimen type	Smear	Culture	tNGS^c^	Endophthalmitis subgroups	Impact of tNGS on therapeutics^d^
1	M	76	OS	VH	Cocci	Negative	*Staphylococcus haemolyticus* 1,057*, Staphylococcus mitis* 42	Post-cataract surgery	Therapeutic regimen unchanged
2	M	66	OD	VH	Bacilli	Negative	*Haemophilus influenzae* 10,400	Post-cataract surgery	Therapeutic regimen unchanged
3	M	76	OD	VH	Cocci	Negative	Negative	Post-cataract surgery	Therapeutic regimen unchanged
4	F	90	OS	AH	A few bacilli	*Streptococcus mitis*	*Staphylococcus aureus* 123	Post-cataract surgery	Therapeutic regimen unchanged
5	M	51	OD	VH	Not tested	Not tested	*Aspergillus niger* 722	Post-vitrectomy	Ocular and systemic antifungal agents
6	M	53	OS	AH	Negative	Not tested	Negative	Post-vitrectomy	False-negative
7	F	70	OS	VH	Not tested	*Mycobacterium abscessus*	*Mycobacterium abscessus* 30,245, *Cytomegalovirus* 91,380	Post-corneal transplant	Amikacin
8	F	80	OD	AH	Abundant cocci	*Streptococcus sanguinis*	*Streptococcus mitis* 82	Post-glaucoma surgery	Therapeutic regimen unchanged
9	F	69	OD	VH	Negative	Negative	Negative	Post-glaucoma surgery	False-negative
10	F	64	OS	VH	Negative	Negative	*Candida albicans* 7	Post-glaucoma surgery	Ocular antifungal agents
11	F	66	OS	AH	Not tested	No tested	*Staphylococcus epidermidis* 1,453	Post-glaucoma surgery	Therapeutic regimen unchanged
12	F	72	OD	AH	Bacilli	Negative	*Streptococcus mitis* 86,846	Post-artificial lens suspension surgery	Therapeutic regimen unchanged
13	F	52	OD	VH	Cocci, a few bacilli	Negative	*Haemophilus influenzae* 183,176	Post-artificial lens suspension surgery	Therapeutic regimen unchanged
14	M	75	OS	AH	A few bacilli	Negative	*Staphylococcus epidermidis* 62,133	Post-intravitreal injection	Therapeutic regimen unchanged
15	M	61	OS	VH	Negative	Negative	*Staphylococcus aureus* 5,612	Endogenous	Ocular and systemic antibacterial therapy
16	M	40	OS	VH	Negative	Negative	*Candida albicans* 43	Endogenous	Ocular and systemic antifungal therapy
17	M	59	OD	AH	Bacilli and cocci	Negative	*Klebsiella pneumoniae* 2,487, *Cytomegalovirus* 40, *Candida albicans* 36	Endogenous	Intravitreal injection of povidone-iodine
18	M	72	OD	AH	Not tested	Negative	*Acinetobacter baumannii* 37,680, *Klebsiella pneumoniae* 3,636, *Stenotrophomonas maltophilia* 2,334	Endogenous	Ocular and systemic antibacterial therapy
19	F	49	OS	VH	Negative	Negative	*Listeria monocytogenes* 214, *Enterobacter cloacae complex* 202, *Staphylococcus aureus* 44	Endogenous	Ocular and systemic antibacterial therapy
20	F	75	OS	VH	Fungal hyphae, a few cocci	Negative	*Aspergillus fumigatus* 1,272	Endogenous	Ocular and systemic antifungal therapy
21	F	51	OD	VH	A few bacilli	Negative	*Klebsiella pneumoniae* 7,050	Endogenous	Ocular and systemic antibacterial therapy
22	F	49	OD	AH	Negative	Negative	*Staphylococcus aureus* 130	Endogenous	Ocular and systemic antibacterial therapy
23	F	67	OS	VH	Negative	Negative	*Aspergillus niger*	Endogenous	Ocular and systemic antifungal therapy
24	F	56	OD	AH	Negative	Not tested	*Aspergillus flavus* 99	Endogenous	Ocular and systemic antifungal therapy
25	M	46	OS	VH	Cocci	Negative	*Staphylococcus aureus* 681	Endogenous	Ocular and systemic antibacterial therapy
26	F	59	OS	VH	Negative	Negative	*Candida parapsilosis* 32	Endogenous	Ocular and systemic antifungal therapy
27	M	49	OS	VH	Not tested	Not tested	*Aspergillus fumigatus* 481	Endogenous	Ocular and systemic antifungal therapy
28	M	22	OD	VH	Not tested	Not tested	Negative	Endogenous	False-negative (mNGS further detected *Chaetomium globosum*)
29	M	51	OD	VH	Negative	Negative	*Aspergillus niger* 421	Trauma	Ocular and systemic antifungal therapy
30	F	64	OD	VH	Few bacilli	Negative	*Streptococcus agalactiae*	Trauma	Therapeutic regimen unchanged
31	M	34	OS	VH	Negative	*Mycelia sterilia* (10 days)	*Pseudomonas aeruginosa* 2,389	Trauma	Intravitreal injection of povidone-iodine
32	M	57	OD	VH	Not tested	Not tested	*Candida albicans* 43	Trauma	Ocular antifungal therapy
33	M	16	OS	AH	Negative	Negative	*Streptococcus pneumoniae* 13,780	Trauma	Therapeutic regimen unchanged
34	F	41	OS	VH	Cocci, a few bacilli	Negative	Negative	Trauma	False-negative
35	M	76	OD	VH	A few bacilli	Negative	*Bacillus cereus* 12,030	Trauma	Antibacterial and anti-inflammatory treatment
36	M	54	OD	VH	Negative	Negative	*Bacillus cereus* 1,446	Trauma	Antibacterial and anti-inflammatory treatment
37	M	49	OS	VH	Cocci	Negative	*Enterococcus faecium* 70, BK polyomavirus 3	Trauma	Therapeutic regimen unchanged
38	M	6	OD	AH	Bacilli	Negative	*Streptococcus pneumoniae* 7,453	Trauma	Therapeutic regimen unchanged
39	M	27	OS	VH	Negative	*Staphylococcus epidermidis*	*Escherichia coli* 6,348, *Staphylococcus epidermidis* 22	Trauma	Therapeutic regimen unchanged
40	M	34	OS	VH	Cocci	*Staphylococcus epidermidis*	*Staphylococcus epidermidis* 184,261, *Escherichia coli* 596, *HBV* 112858	Trauma	Therapeutic regimen unchanged
41	M	43	OD	VH	Bacilli	*Citrobacter koseri*	*Citrobacter koseri* 291,061	Trauma	Therapeutic regimen unchanged
42	M	36	OS	VH	Negative	Negative	*Aspergillus flavus* 8,069	Trauma	Ocular and systemic antifungal therapy
43	F	55	OD	VH	Fungal hyphae	*Mycelia sterilia* (14 days)	Negative	Trauma	False-negative

a, M = male, F = female; b, OS = left eye, OD = right eye; c, the numbers of reads follow the species name; the detection of viruses in some cases was not assessed further as viruses are not associated with endophthalmitis; d, cases were highlighted in case of significant impact of tNGS on clinical decision-making.

### 3.2 Performance of the conventional smear and culture methods

Thirty-six specimens were smeared, with positive results observed in 20 specimens (positivity rate, 20/36, 55.6%). Among these positive smear results, 12 specimens detected bacilli, 10 detected cocci, 2 detected fungi, and polymicrobial infections were reported in 4 specimens (Table 2). Of the 36 specimens subjected to microbiological culture, 8 yielded positive results (positivity rate, 8/36, 22.2, 95% CI: 10.1–39.2%), with no polymicrobial infections identified (Table 2). Notably, for Cases No. 31 and 43, Mycelia sterilia was detected after 10 and 14 days of culture, respectively (Table 2), indicating a relatively long culture turnaround time.

Among the 34 specimens subjected to both smear and culture, 12 (12/34, 35.3%) were double-negative, and 5 were double-positive. Of the 5 double-positive specimens, 1 showed inconsistent findings: smear reported bacilli, whereas culture identified *Streptococcus mitis* (Case No. 4, Table 2). These findings indicate that the application of alternative methods is warranted to enhance pathogen identification.

### 3.3 Performance of tNGS

All 43 specimens were subjected to tNGS, which was designed to target a predefined panel of 207 clinically relevant pathogens (Table 1). Microbes were detected in 37 specimens, corresponding to a positivity rate of 86.0% (95% CI: 72.1–94.7%), with a turnaround time of < 24 h for all positive detections. Specifically, 20 specimens were identified as having a single bacterial infection, 11 as having a single fungal infection, 5 as having a mixed bacterial infection, and 1 as having a mixed bacterial-fungal infection (Case No. 17). Notably, for Cases No. 39 and 40, tNGS further detected *Escherichia coli*, in addition to *Staphylococcus epidermidis* that was reported by culture.

Importantly, tNGS detected microbes in 11 of the 12 specimens (91.7%) that were negative for both smear and culture (Table 2), suggesting the advantage of this method in enhancing pathogen identification.

### 3.4 Comparison between tNGS and culture

A total of 36 specimens were detected by both culture and tNGS, with 7 specimens being double-positive and 3 being double-negative (Table 3). Among the 7 double-positive cases, inconsistent results between the two methods were observed in 3 cases (3/7, 42.9%), including Cases No. 4, 8, and 31 (Table 2). Specifically, for Case No. 4, culture identified *Streptococcus mitis*, whereas tNGS detected *Staphylococcus aureus*. For Case No. 8, culture detected *Streptococcus sanguinis*, while tNGS identified *Streptococcus mitis*. The application of tNGS for the two cases facilitated timely antibacterial treatment. Notably, for Case No. 31, tNGS reported *Pseudomonas aeruginosa*, whereas culture detected *Mycelia sterilia* after a 10-day incubation period (Table 2).

**Table 3:** Results for the 36 samples detected by both culture and tNGS.

Results	tNGS (+)	tNGS (−)	*p* value	*Kappa*
Culture (+)	7	1	<0.001^a^	
Culture (−)	25	3		−0.009^b^

a, by McNemar test; b, *p* = 0.887.

On the other hand, consistent findings between culture and tNGS were observed in 4 of the 7 double-positive cases (4/7, 57.1%), specifically Cases No. 7, 39, 40, and 41. For Case No. 7, culture and tNGS detected *Mycobacterium abscessus*. For Case No. 41, both methods identified *Citrobacter koseri*. For Cases 39 and 40, tNGS further detected polymicrobial infections, i.e., *Escherichia coli* and *Staphylococcus epidermidis* (the latter detected by culture) (Table 2).

Notably, tNGS detected microbes in 25 of the 28 culture-negative specimens (89.3%) (Table 3). Among the paired specimens, positivity rate of tNGS was 88.9% (32/36, 95% CI: 73.9–96.9%) Statistical analysis by McNemar’s test demonstrated that the positivity rate of tNGS was significantly higher than that of culture (*p* < 0.001, Table 3), with the difference in positivity rates being 66.7% (95% CI: 44.4–80.6%). Poor agreement between tNGS and culture for binary positivity was observed (Kappa = −0.009, *p* = 0.887, Table 3). These findings suggest the added value of tNGS in enhancing pathogen identification in patients with suspected endophthalmitis.

However, for Case No. 43, culture detected *Mycelia sterilia*, whereas tNGS yielded negative results (Table 2).

We next evaluated whether the type of specimens could differentially affect the performance of culture and tNGS. Interestingly, among the 9 AH samples subjected to both culture and tNGS, all 9 (100%) were tNGS-positive, whereas only 2 specimens (2/9, 22.2%) were culture-positive (Table 4). For the 27 VH specimens analyzed by both methods, 23/27 (85.2%) were tNGS-positive, and 6 of 27 (22.2%) were positive for culture. McNemar’s test revealed a statistically significant difference in positivity rates between the two methods (*p* < 0.001, Table 5).

**Table 4:** The 9 AH specimens detected by both culture and tNGS.

Result	tNGS	Culture
Positive	9 (100%)	2 (22.2%)
Negative	0^a^	7

a, statistical analysis was not performed given the “0” value for the group.

**Table 5:** The 27 VH specimens detected by both culture and tNGS.

Result	tNGS	Culture	*p*
Positive	23 (85.2%)	6 (22.2%)	<0.001^a^
Negative	4	21	

a, McNemar test.

### 3.5 Performance of tNGS for Endophthalmitis subgroups

Furthermore, we observed that tNGS detected microbes in 13 of 14 cases of endogenous endophthalmitis, in 13 of 15 cases of post-traumatic endophthalmitis, and in 11 of 14 cases of post-operative endophthalmitis. Fisher’s exact test revealed no statistical difference for the positive rates across the subgroups (*p* = 0.666, Table 6).

**Table 6:** Results of tNGS for endophthalmitis subgroups.

Subgroups	tNGS (+)	tNGS (−)	*p* value
Endogenous endophthalmitis	13	1	0.666^a^
Post-traumatic endophthalmitis	13	2	
Post-operative endophthalmitis	11	3	

a, by Fisher’s exact test.

### 3.6 Impact of tNGS on therapeutic strategy selection

The impact of tNGS on clinical therapeutic strategies was further analyzed. As shown in Table 2, for patients with post-traumatic and post-operative endophthalmitis, the identification of bacterial species by tNGS generally did not result in changes to the therapeutic regimen. Exceptions were observed in infections caused by uncommon pathogens, such as *Mycobacterium abscessus* in Case No. 7 (identified also by culture, 7 days of incubation) and *Bacillus cereus* in Cases No. 35 and 36. In these specific cases, tNGS-facilitated prompt pathogen identification led to adjustments in clinical therapeutic strategies, contributing to the control of infection (Table 2).

For endogenous endophthalmitis, the identification of bacterial species by tNGS influenced clinical decision-making, specifically guiding the systemic administration of antibacterial agents (Table 2). Importantly, the clinical significance of tNGS was most highlighted in cases with fungal or mixed infections, including Cases No. 5, 10, 16, 17, 20, 23, 24, 26, 27, 29, 32, and 42 (Table 2). The administration of topical and/or systemic antifungal agents in these cases contributed to the control of infections.

## 4 Discussion

**Figure 1:**
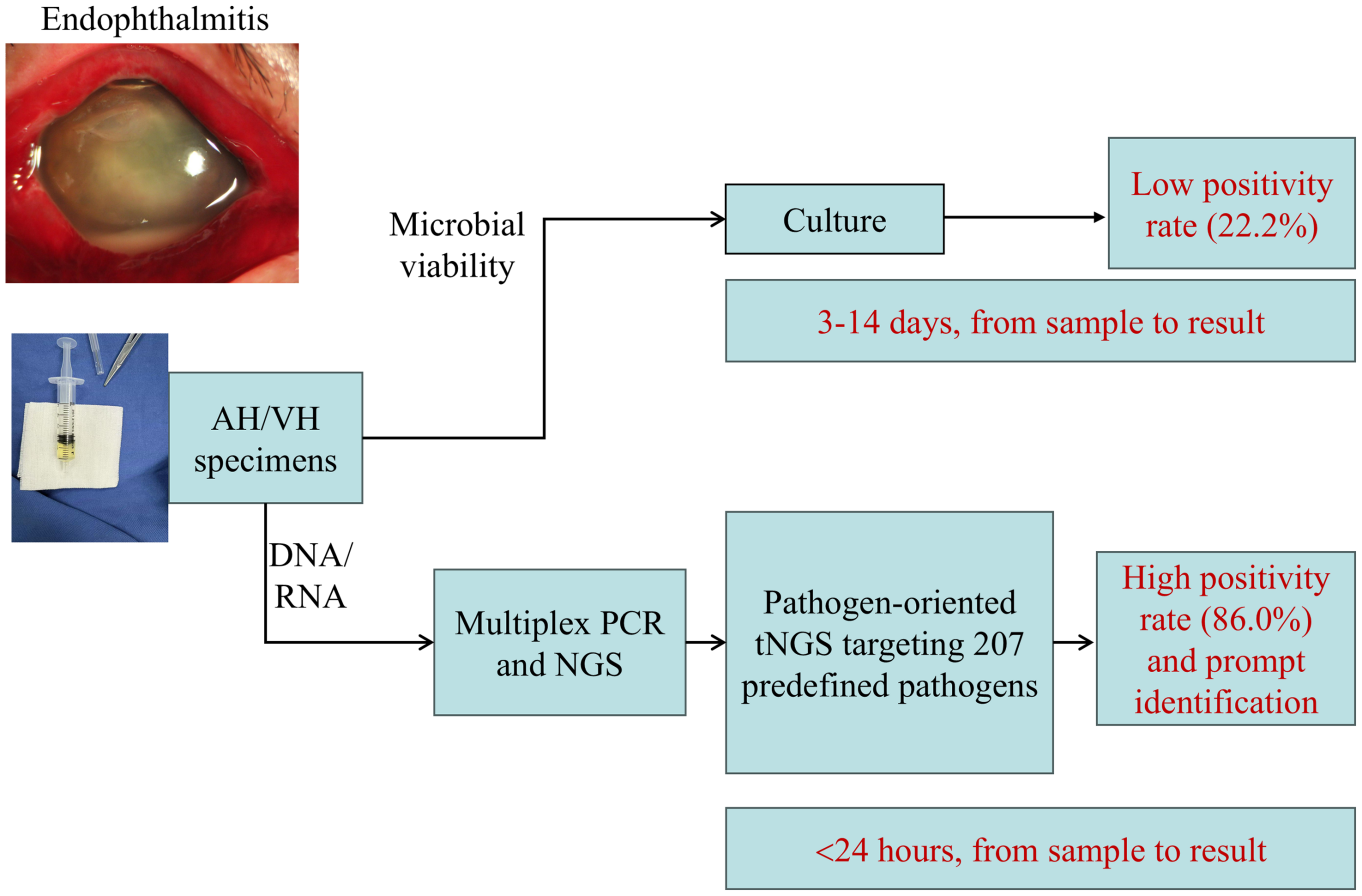
tNGS as an added diagnostic approach for pathogen identification in endophthalmitis. Clinical specimens, aqueous humour (AH) or vitreous humour (VH), were detected by culture and the advanced tNGS tool, showing substantially reduced detection turnaround time and significantly increased detection yield for tNGS.

The diagnosis of endophthalmitis, an ocular emergency potentially resulting in vision loss, relies on clinical manifestations and laboratory diagnostic techniques. Although the culture of VH or AH specimens is regarded as the gold standard for diagnosis, it exhibits relatively low sensitivity. More advanced molecular tools, such as targeted or nontargeted NGS, are increasingly being employed in clinical practice, thereby facilitating rapid and accurate pathogen identification (20). To date, NGS targeting the bacterial 16S rRNA gene and fungal ITS region (7, 9, 10), as well as unbiased mNGS (21), have been evaluated in patients with suspected endophthalmitis. In the present study, the detection performance of amplicon sequencing-based tNGS, designed to simultaneously detect a predefined panel of 207 pathogens, was investigated for the first time in patients with suspected endophthalmitis (Figure 1).

### 4.1 tNGS shortened the detection turnaround time

The conventional culture method has multiple limitations, including a relatively long turnaround time, with 3–4 days for bacterial detection and an additional 2–3 days for fungal identification (16). This limitation can be further exacerbated by infections with fastidious microorganisms and/or growth interference in cases of polymicrobial infections. Indeed, in the present study, the turnaround time from specimen collection to culture result ranged from 3 to 14 days, most notably 10 days for Case No. 31 and 14 days for Case No. 43 (both detecting *Mycelia sterilia*). The turnaround time for tNGS was less than 24 h in all cases (Figure 1). Particularly, for Case No. 7, while both culture and tNGS detected *Mycobacterium abscessus*, the turnaround time for tNGS was reduced from 7 days (for culture) to within 24 h, contributing to early shift to amikacin treatment (Table 2). Therefore, the application of tNGS has the potential to greatly expedite pathogen identification, potentially enabling more rapid clinical decision-making and targeted antimicrobial therapy in ocular emergencies, particularly for endophthalmitis.

### 4.2 tNGS demonstrated a higher positivity rate, facilitated the detection of fungal and polymicrobial infections, and seemed to exhibit no diagnostic bias across different specimen types or endophthalmitis subgroups

The traditional culture method exhibits relatively low detection sensitivity, attributed to multiple factors such as small specimen volumes, prior antibiotic administration and/or infection with fastidious or slow-growing microbes (10, 16, 22). Consistent with previous reports (16), the positivity rate of microbiological culture was low in the present study, accounting for 22.2% (8/36). In contrast, tNGS demonstrated a significantly higher positivity rate, reaching 86.0% (37/43). More importantly, tNGS successfully detected microbes in 25 of the 28 culture-negative specimens (89.3%), thereby improving detection yields (Figure 1). These findings may highlight the strengths and added value of tNGS in enhancing pathogen identification in patients with endophthalmitis.

tNGS also facilitated the detection of polymicrobial infection, whereas no coinfections could be identified by culture. While it is relatively difficult, at this stage, to ascertain whether the microbes detected by tNGS were true agents causing the infection and whether the cases were true coinfections, the application of tNGS did contribute to better clinical decision-making in particular cases (e.g., No. 17, 18, and 19). For Case No. 17, the identification of *Klebsiella pneumoniae* and *Candida albicans* coinfection led to adjustments in the therapeutic strategy, specifically intravitreal injection of povidone-iodine, contributing to the control of infection (Table 2). For Cases 18 and 19, the detection of co-bacterial infection by tNGS in these endogenous cases led to ocular and systemic antibacterial therapy (Table 2). For Cases No. 39 and No. 40, in addition to *Staphylococcus epidermidis* detected by culture, tNGS further identified *Escherichia coli*. In the specific cases, the application of tNGS, however, had no impact on clinical decision-making (Table 2). It is hence challenging to verify whether tNGS findings represent true coinfection or colonization/contamination.

The tNGS-facilitated detection of *Mycobacterium abscessus* in Case No.7 and *Bacillus cereus* in Cases No. 35 and 36 contributed to control of infection through adjustments in therapeutic regimens. In Case No. 7, a shift in the therapeutic regimen from empirical antibacterial agents to amikacin effectively controlled mycobacterial infection. In the latter two cases, considering the inflammatory characteristics of *Bacillus cereus* infections (23), the administration of combined antibacterial and anti-inflammatory therapy resulted in favorable clinical outcomes.

Notably, tNGS detected fungal or polymicrobial infections in 12 cases (Cases 5, 10, 16, 17, 20, 23, 24, 26, 27, 29, 32, and 42; Table 2). Fungal endophthalmitis poses a significant clinical challenge due to its insidious presentation, long incubation period, and propensity for misdiagnosis (2). As clinical therapeutic strategies, including the duration of systemic antifungal therapy, are heavily dependent on the identified fungal species, the ability of tNGS to identify fungi across all endophthalmitis subtypes has profound implications for clinical decision-making. Specifically, the timely administration of tailored topical and/or systemic antifungal regimens, informed by the prompt tNGS results, was instrumental in achieving successful control of infection in the aforementioned cases.

It is known that VH specimens, obtained via invasive surgical procedures, are generally associated with a higher culture positivity rate than AH specimens (10). For instance, Low et al. (24) reported a culture positivity rate of 78.3% (18/23) among 23 specimens, including 22 VH samples. However, in the present study, no difference in culture positivity rates was observed between VH and AH specimens, with both yielding a positivity rate of 22.2%. This discrepancy is likely attributed to the small sample sizes assessed (i.e., 9 AH and 27 VH specimens). Interestingly, the use of AH specimens did not seem to compromise the positivity rate of tNGS. Indeed, all 9 AH specimens were tNGS-positive, while 23 of 27 (85.2%) VH specimens were tNGS-positive. Interestingly, Qian et al. (25) recently reported a slightly higher mNGS sensitivity for VH samples (92.2%) than for AH samples (85.4%). It is likely that the targeted enrichment feature of tNGS, endowed with higher detection sensitivity than mNGS (12), may mitigate potential performance bias across different specimen types. However, the small sample size assessed in the present study, e.g., only 9 AH specimens, may lead to biased estimates of positivity rates between AH and VH. Further studies are required to investigate whether AH specimens, which are less invasive and more convenient to obtain, are preferred when using tNGS for pathogen detection in suspected endophthalmitis.

The results also seemed to support that the performance of tNGS did not differ significantly among post-traumatic, endogenous, and post-operative endophthalmitis. The positivity rate of culture was shown to be influenced by the infection source. Indeed, Duan et al. (4) reported a higher culture positivity rate in endogenous endophthalmitis compared with that in post-operative endophthalmitis. Compared with the culture method, based on microbial viability detection, tNGS detects pathogen nucleic acids and is expected to be less likely affected by prior antibiotic administration (26).

Herein, we observed that 3 of the 7 specimens positive for both culture and tNGS exhibited inconsistent findings. Specifically, Cases No. 4 (AH specimen), 8 (AH specimen) and 31 (VH specimen), were post-traumatic or post-operative endophthalmitis cases. For Cases No. 4 and No. 8, tNGS and culture identified different bacterial species within the same genus (i.e., *Streptococcus*). In the two cases, the use of tNGS did not alter the clinical management strategy (i.e., empirical antibacterial treatment) (Table 2). It is rather challenging to confirm which result represented the true infection, at this stage. For Case No. 31, tNGS detected *Pseudomonas aeruginosa* within 24 h, whereas culture identified *Mycelia sterilia* following a 10-day incubation period (Table 2). Guided by the tNGS results, antibacterial treatment was initiated first. Subsequently, based on the culture findings, intravitreal injection of povidone-iodine was added, and the infection was controlled. Similarly, it remains difficult to determine the causative agent for this specific case.

### 4.3 Comparison of tNGS with other NGS approaches

To date, various sequencing techniques have been applied in the diagnosis of suspected endophthalmitis, including mNGS (21), Illumina/Nanopore whole-genome sequencing (24), and NTS (16). Li et al. (16) investigated the performance of NTS, which is based on the amplification of the bacterial 16S rRNA gene and fungal ITS1/2 regions, and reported a positivity rate of 86.05%. Although NTS offers advantages such as long-read sequencing and real-time analysis, it may also detect nonpathogenic microbes, particularly those of the ocular microbiome (16, 27, 28). This could potentially confound clinical decision-making. In addition, unbiased mNGS has facilitated pathogen detection in patients with suspected endophthalmitis, with a reported positivity rate ranging from 88.89 to 90.63% (21, 29). However, mNGS has several limitations, including higher costs and potential background signal interference (e.g., human and microbiota-derived reads) (22).

Compared with the aforementioned sequencing tools, the tNGS approach employed in the present study, based on ultra-multiplex PCR-mediated enrichment of pathogen-specific genes and amplicon sequencing, exhibits some advantages, including higher positivity rates and efficiency, lower cost, shorter turnaround time, and customizability. These characteristics could render it a suitable method for the detection of both pathogens and antibiotic resistance genes (30).

However, despite its wide coverage of 207 predefined pathogens, the pathogen-oriented tNGS failed to detect pathogens in 6 cases (No. 3, 6, 9, 28, 34, and 43). Among the 6 cases, 3 were positive for smear and/or culture (Cases No. 3, 34, and 43; Table 2), reflecting limitations of the tNGS approach. This detection failure may be attributed to either the absence of the causative pathogen in the tNGS detection panel, the quality of specimens collected, or simply false-negative errors. Specifically for Case No. 28, mNGS further detected infection with *Chaetomium globosum*, which is not included in the tNGS panel (Tables 1, 2). As the panel design itself may apparently shape the spectrum of detected organisms, cautions should be taken to interpret tNGS-negative results. For Cases No. 3, 6, 9 and 34, tNGS failed to identify pathogens. Empirical antibacterial treatment in these cases contributed to control of infection. Therefore, it is proposed that tNGS be used in conjunction with conventional diagnostic methods (e.g., microbial culture) in clinical practice, and that unbiased mNGS be further employed for rare cases that are negative for both culture and tNGS.

A recent Chinese expert consensus on the application of mNGS in ocular infectious diseases states that negative mNGS results should not be used as a basis to completely rule out ocular infectious diseases (31). Considering the risk of false-negative results, similar caution is required when interpreting negative tNGS findings.

## 5 Conclusion

The higher detection yield and shorter turnaround time of tNGS render it a valuable tool for etiological identification in endophthalmitis. We propose the routine incorporation of tNGS as an adjunctive approach to the conventional microbial culture method, with the aim of facilitating and expediting pathogen detection and promoting tailored precision medicine.

However, the present study has inherent limitations, particularly its retrospective nature. This retrospective nature precluded more standardized specimen collection, likely introducing selection bias. Such bias could be further exacerbated by the relatively small sample size enrolled in the study (i.e., 43 cases of suspected endophthalmitis). On the other hand, given that all enrolled cases were clinically diagnosed with endophthalmitis and no non-infectious control group was included in this study, we were unable to calculate the positive and negative predictive values of tNGS (32). Future investigations incorporating both patients with endophthalmitis and individuals without intraocular infection are warranted to comprehensively evaluate the diagnostic performance of tNGS. In addition, the limitation of tNGS includes the requirement of special lab facilities and higher cost, relative to conventional culture.

## Data Availability

The raw tNGS sequencing data generated in this study contain sensitive patient clinical information and cannot be publicly deposited or shared due to ethical restrictions imposed by the institutional ethics committee. De-identified datasets are available from the corresponding author upon reasonable request with approved ethical clearance.
